# Early prepubertal cyclophosphamide exposure in mice results in long-term loss of ovarian reserve, and impaired embryonic development and blastocyst quality

**DOI:** 10.1371/journal.pone.0235140

**Published:** 2020-06-23

**Authors:** Sujith Raj Salian, Shubhashree Uppangala, Aswathi Cheredath, Fiona D’Souza, Guruprasad Kalthur, Vinod C. Nayak, Richard A. Anderson, Satish Kumar Adiga

**Affiliations:** 1 Department of Clinical Embryology, Kasturba Medical College, Manipal, Manipal Academy of Higher Education, Manipal, India; 2 Department of Forensic Medicine, Kasturba Medical College, Manipal, Manipal Academy of Higher Education, Manipal, India; 3 MRC Centre for Reproductive Health, University of Edinburgh, Edinburgh, United Kingdom; Universiteit Utrecht, NETHERLANDS

## Abstract

**Background:**

Due to improved treatment, there is an increasing focus on the reproductive potential of survivors of childhood cancer. Cytotoxic chemotherapy accelerates the decline in the number of primordial follicles within the mammalian ovary at all ages, but effects on the developmental potential of remaining oocytes following prepubertal cancer treatment are unclear.

**Objectives:**

To investigate whether cyclophosphamide (CY) exposure in the prepubertal period in female mice influences ovarian function and the functional competence of oocytes in adulthood.

**Methods:**

This study used Swiss albino mice as the experimental model. Female mice were treated with 200 mg/kg CY on either postnatal day 14 (CY14), 21 (CY21) or 28 (CY28) i.e at a prepubertal and 2 young postpubertal ages. At 14 weeks of life, ovarian function, functional competence of oocytes, and embryo quality were assessed.

**Results:**

The number of primordial follicles decreased significantly in CY14 and CY21 groups compared to control (p < 0.01). The number of oocytes from superovulated was 8.5 ± 1.4, 24.1 ± 2.9 and 26.8 ± 2.1 in CY14, CY21 and CY28 respectively which was significantly lower than control (50.2 ± 3.2; p < 0.001). *In vitro* culture of CY14 embryos demonstrated only 55.4% blastocyst formation (p < 0.0001) and reduced ability of inner cell mass (ICM) to proliferate *in vitro* (p < 0.05) at 120 and 216 h post insemination respectively. On the other hand, ICM proliferation was unaltered in 2 young postpubertal ages.

**Conclusion:**

Our results indicate long-term effects on the developmental competence of oocytes exposed to CY in early but not adult life. These data provide a mechanism whereby long-term fertility can be impaired after chemotherapy exposure, despite the continuing presence of follicles within the ovary, and support the need for fertility preservation in prepubertal girls before alkylating agent exposure.

## Introduction

Childhood cancer survival has improved greatly over the past five decades, and due to improved treatment modalities, about 70–80% of children with cancer will be cured. Many childhood cancer survivors are now entering their reproductive age [1]. However, certain types of cancer treatments can compromise ovarian function in children, notably high doses of alkylating agents and radiotherapy [2–4].

Cyclophosphamide (CY) is an alkylating chemotherapeutic agent commonly used to treat solid and haematological malignancies, and autoimmune disorders. The primary target of CY in murine ovaries is the growing follicles with additional direct and indirect effects on primordial follicles [5–7]. Oocytes within primordial follicles exist in a relatively quiescent state, and hence may be relatively resistant to antimitotic agents [8], but the effects of alkylating agents are independent of the cell cycle. On the other hand, recent studies have shown that CY is also capable of damaging the primordial follicles in murine ovaries [7,9]. Rodents exposed to CY in pregnancy (i.e. before primordial follicle formation) also showed a reduced number of primordial follicles and increased follicle growth activation in the offspring [10].

The ovaries have a finite number of primordial follicles at birth, with this constituting the ovarian reserve. The irreversible decline in the number of primordial follicles within a prepubertal ovary starts as soon as follicles begin to form and this process continues till menopause. The decline in the primordial follicle pool is accelerated when girls and women undergo cytotoxic chemotherapy, leading to premature ovarian insufficiency (POI) and infertility [11,12]. It has been shown that cancer chemotherapy significantly damages prepubertal ovaries, causes follicle maturation arrest, induces stromal fibrosis, and decreases the number of oocytes. These effects have been seen with the administration of just a single dose of alkylating chemotherapy agent [13].

While chemotherapy treatment can induce a complete loss of ovarian function (POI), there is also evidence that women are more likely to report infertility after both adult and childhood cancer treatment, even when there is ongoing ovarian function [14,15]. This may indicate damage to follicles and oocytes that is insufficient to cause their loss, but may reduce their developmental competence. The prepubertal ovary contains populations of follicles and oocytes with abnormal morphology that are not found in the adult ovary, and isolated follicles show different growth characteristics [16] and in mouse, the prepubertal ovarian environment undergoes significant transformation before and on entering puberty [17]. Thus, the prepubertal period is a time of ongoing maturation towards adult ovarian function, rather than just relative quiescence. Additionally, younger oocytes may have greater capacity for DNA repair and therefore be able to respond to toxic insults to a greater extent than older ones [18].

Therefore, understanding the impact of chemotherapeutic agents on prepubertal ovaries is important in addressing the risk of abnormal reproductive outcomes in childhood cancer survivors in their adult life. The long-term nature of these studies and the difficulties in studying oocyte function in the human require the use of animal models. Using the mouse, we have examined the functional competence of female germ cells exposed to CY, comparing early prepubertal exposure with exposure in other groups. When prepubertal (14 day old) mice were exposed to CY, our results indicate impairment in ovarian function and gamete quality leading to poor embryonic development.

## Materials and methods

### Animals and experimental design

All experiments and animal handling were conducted in accordance with the institutional guidelines for animal experimentation after obtaining prior approval from the Institutional Animal Ethics Committee (Kasturba Medical College & Kasturba Hospital Institutional Ethics Committee, approval #IAEC/KMC/61/2016 & IAEC/KMC/21/2018). The animals were housed and maintained in controlled conditions of 23 ± 2°C, 12 h light-dark cycle, 50 ± 5% humidity, and were fed with standard diet and water *ad libitum*.

The experimental design involved treatment of a total of one hundred and three healthy female siblings from 24 litters, where ninety-one siblings were divided into groups for CY exposure on either postnatal day 14, postnatal day 21 or postnatal day 28 (CY14, CY21 & CY28 respectively), and twelve siblings were used as control. The CY animals were weighed on either day 14 (N = 54), day 21 (N = 21), or day 28 (N = 16) and administered a single intra-peritoneal injection of 200 mg/kg CY (C7397, Sigma Aldrich, USA), the control mice received an equal volume of phosphate buffer saline (PBS) on day 14, 21 and/or 28 and grouped as randomized control (N = 12). The CY exposed female mice were monitored every week for fur loss, change in body weight and survival. A recovery period post-treatment was decided on the ability of the animal to overcome the CY dose in terms of fur loss, gain in body weight and survival. A minimum body weight of 20 g was set as standard before considering them as fit for ovarian stimulation. Therefore, at 14 weeks of life, irrespective of post-treatment interval, the surviving animals were superovulated as described below.

### Ovarian stimulation

Female mice were superovulated by the intra-peritoneal injection (5 IU) of pregnant mare serum gonadotropin (PMSG, Sigma Aldrich, USA) followed by the administration of 10 IU of human chorionic gonadotropin (hCG, Eutrig-HP) after 48 h. The animals were weighed and euthanized 13 h post hCG by cervical dislocation. Oocyte Cumulus Complexes (OCCs) were collected from the oviduct.

### Ovarian histology

**Fig 1:**
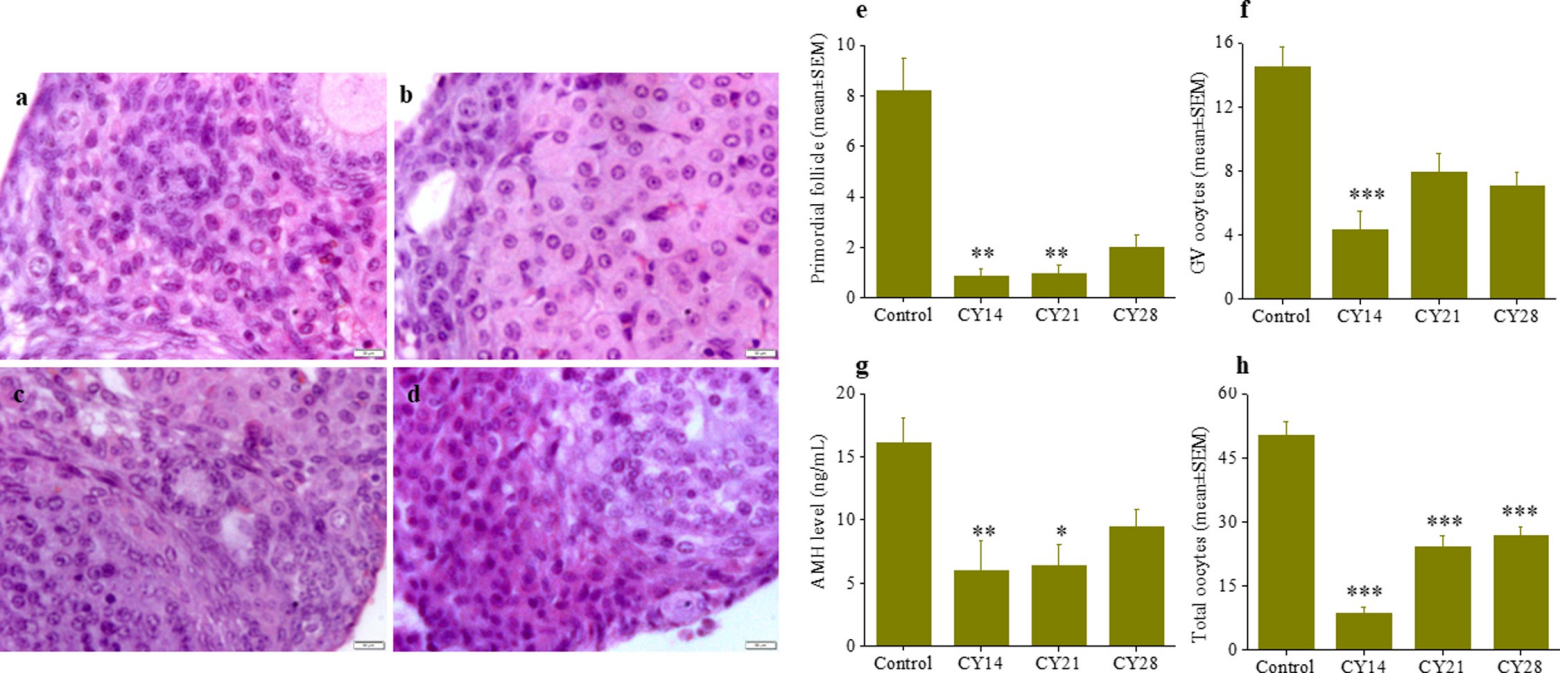
**Prepubertal CY exposure on ovary at 14 weeks of life (a-d) Representative images of histological examination: control, CY14, CY21 & CY28, respectively**. The scale bar represents 50 μm. (**e**) Mean (± SEM) number of primordial follicle per ovary section examined, in control (N = 9), CY14 (N = 7), CY21 (N = 4) & CY28 (N = 6), **p < 0.01 vs. control. (f) Number of GV oocytes retrieved from stimulated ovaries, in control (N = 6), CY14 (N = 18), CY21 (N = 16) & CY28 (N = 14), ***p < 0.001 vs. control. (g) AMH level in blood serum (N = 5 per group), *p < 0.05 & **p < 0.01 vs. control. (h) Mean (± SEM) total number of oocytes, post superovulation, in control (N = 6), CY14 (N = 18), CY21 (N = 16) & CY28 (N = 14) females, ***p < 0.001 vs. control.

Extracted ovaries were weighed and either fixed in Bouin’s solution for histological analysis. The other ovary from each animal was gently teased (cortical region) under a stereomicroscope using fine needles to release GV oocytes from the secondary/tertiary follicles. The fixed ovaries were then paraffin-embedded and cut into 5 μm sections on slides. After deparaffinization and rehydration, the sections were stained with Haematoxylin and Eosin (H&E) and examined for the number of primordial, primary, secondary and antral follicles under light microscopy. Only those follicles in which the nucleus of the oocyte was clearly visible were scored. Primordial follicles had an intact oocyte surrounded by a single layer of flattened squamous follicular cells, primary follicles had an intact oocyte surrounded by a single layer of cuboidal follicular or supporting cells, secondary follicles had an intact oocyte surrounded by multiple layers of follicular or supporting cells and antral follicles had an intact oocyte with multiple layers of granulosa cells, a basal lamina, a theca interna and a theca externa with fluid-filled cavity. The histological analysis was performed by assessing the follicle count and imaging. A minimum of 4 ovaries were taken per group and the follicle data are presented in Figs 1 and S1.

### Hormone measurements

Antimullerian hormone (AMH) levels in serum were measured by Electrochemiluminescence Immunoassay (ECLIA) using a COBAS e411 analyzer using the specified kit with a detection range of 0.01–23 ng/mL (Roche, Mannheim, Germany). The values are expressed as ng/mL serum.

### Mitochondrial activity in metaphase II oocytes

The mitochondrial activity was estimated as described earlier [19]. Briefly, the denuded metaphase II (MII) oocytes were washed and incubated with freshly prepared 1 μg/mL 5,50,6,60- tetrachloro-1,10,3,30-tetraethylbenzimidazolylcarbocyanine iodide (JC-1, T3168, Molecular probes, Life technologies, USA), at 37°C for 30 min. The oocytes were later washed in DMEM medium (supplemented with 1mg/mL of bovine serum albumin) and imaged for JC-1 monomers and JC-1 aggregates detection, using a fluorescence microscope (Imager-A1, Zeiss, Gottingen, Germany). The ratio of orange and green fluorescence was calculated using ImageJ software (National Institute of Health, Bethesda, Maryland, USA).

### *In vitro* fertilization and preimplantation embryo development

*In vitro* fertilization and embryo culture were carried as described earlier [20]. OCCs from the oviduct were released into pre-warmed Earl’s balanced salt solution (EBSS, E2888, Sigma Aldrich, USA) supplemented with 0.1% bovine serum albumin (BSA). Cumulus-intact oocytes were inseminated with capacitated spermatozoa (final concentration: 3–5×10^5^ spermatozoa/mL) collected from the healthy males. The gametes were co-incubated for 12 h at 37°C in 5% CO_2_. The oocytes were then washed in Potassium Simplex Optimization Medium (KSOM AA), and classified as normally fertilized (having 2 pronuclei and 2 polar bodies) or abnormal (no pronuclei, dead or fragmented). Fertilized oocytes were transferred to fresh KSOM, and cultured until the blastocyst stage. Their developmental potential was assessed under an inverted microscope (IX73, Olympus, Japan) at regular intervals to assess 2-cell, 4-cell, morula and blastocyst rate. For total cell number (TCN), the expanded and hatched blastocysts were fixed in 4% paraformaldehyde for 1 h. The blastocysts were then washed 3 times in phosphate buffered saline (PBS) and the nucleus stained using 4 μg/ml DAPI. They were transferred to clean microscopic slides and total cell number per blastocysts was documented under fluorescent microscope (Carl Zeiss, Gottingen, Germany).

### Inner Cell Mass (ICM) outgrowth assay

Ability of the blastocyst to attach and proliferate post-implantation *in vitro* was assessed using ICM outgrowth assay. *In vitro* derived blastocysts appearing morphologically normal with expanded blastocoel at 120 hour-post insemination (hpi) were selected for ICM outgrowth assay [21,22]. Briefly, multi-well dishes (D7039, Sigma Aldrich, USA) were pre-coated with gelatin (0.1%) for 30 min at room temperature. Excess gelatin was removed and the dishes were air dried. Individual blastocysts at 120 hpi were transferred into each well containing 500 μL Dulbecco’s Modified Eagle Medium (DMEM) 20% supplemented with fetal calf serum (FCS). The embryos were cultured *in vitro* for an additional extended period of 96 h i.e. till 216 hpi. The medium was changed after 48 h of culture and proliferation of inner cell mass and trophectoderm was monitored under an inverted phase contrast microscope (IX73, Olympus, Japan) at the end of culture. Based on morphology and size of the outgrowths, ICM outgrowths (IO) were graded as completely developed ICM outgrowth (CIO), large ICM outgrowth (LIO), small ICM outgrowth (SIO), and no ICM outgrowth (NIO), as described earlier [21]. The ICM and trophectoderm (TE) area were quantified using ProgRes CapturePro (version 2.7.7, Jenoptik, Germany) and presented.

### Statistical analysis

All data are presented as mean±SEM, and analysed using either by One Way Analysis of Variance (ANOVA) for data normally distributed, or by Kruskal-Wallis test followed by Dunn’s test if not normally distributed. Percentage data for preimplantation embryo development and ICM outgrowth assay was analyzed using Chi-square test, all tests performed using GraphPad InStat 3.0 statistical package (GraphPad Inc., USA). All the graphs were plotted using Origin 8.0 (Origin Lab Corporation, Northampton, MA, USA).

## Results

### Effect of prepubertal CY exposure on survival

The intra-peritoneal administration of CY at 200 mg/kg body weight resulted in reduced survival, with the effect reducing with increasing age at treatment [control: 100% survival vs. CY14: 61.1% (p < 0.05), CY21: 76.2%, CY28: 87.5%]. Survival was monitored up to 14 weeks of life (Table 1). The general health of the animals, in terms of weight of various organs, did not change significantly except the liver weight in CY14 (p < 0.01) and CY21 (p < 0.05). Furthermore, the ovarian weight was significantly lower in all CY groups (p < 0.01 vs. control) and the uterine weight declined significantly in CY14 and CY28 in comparison to control (p < 0.001) (S1 Table).

**Table 1:** Effect of prepubertal CY exposure on body weight and survival.

Group	Females (N)	Body weight in g ± SEM (survival) at different weeks of life					
		2 weeks	3 weeks	4 weeks	5 weeks	10 weeks	14 weeks
**Control**	12	6.1 ± 0.2	9.4 ± 0.3	14.0 ± 0.5	18.3 ± 0.5	26.5 ± 0.6	27.9 ± 0.4
		(100)	(100)	(100)	(100)	(100)	(100)
**CY14**	54	6.6 ± 0.2	7.0 ± 0.2 ^c^	9.7 ± 0.3 ^c^	11.6 ± 0.5 ^c^	15.9 ± 0.6 ^c^	24.6 ± 0.6 ^b^
		(100) ■	(90.7)	(88.9)	(85.2)	(72.2)	(61.1) ^a^
**CY21**	21	6.2 ± 0.2	10.3 ± 0.4	11.4 ± 0.5 ^a^	14.0 ± 0.9 ^b^	19.3 ± 0.9 ^b^	24.7 ± 0.6 ^b^
		(100)	(100) ■	(100)	(100)	(80.9)	(76.2)
**CY28**	16	6.1 ± 0.2	9.1 ± 0.2	14.0 ± 0.3	15.3 ± 0.7	19.8 ± 1.0 ^a^	25.2 ± 0.6 ^a^
		(100)	(100)	(100) ■	(93.8)	(87.5)	(87.5)

■ Indicates the time of CY injection (single dose of 200 mg/Kg body weight, i.p.).

^**a**^p < 0.05,

^**b**^p < 0.01,

^**c**^p < 0.001 vs. control.

### Prepubertal CY exposure reduces primordial follicle numbers and Antimullerian hormone (AMH) level

Examination of ovaries from CY treated animals showed significant alterations in the ovarian histology (Fig 1A–1D). The number of primordial follicles was decreased significantly in CY14 and CY21 groups (Figs 1E and S1) compared to control (p < 0.01). There were no differences in the number of primary or antral follicles between groups, although the number of secondary follicles was significantly lower in CY14 (p < 0.01, S1 Fig) and serum AMH was significantly lower in CY14 (p < 0.01) and CY21 (p < 0.05) in comparison to control (Fig 1G). Furthermore, the number of germinal vesicle (GV) oocytes retrieved from the secondary/tertiary follicles in the stimulated ovaries was significantly lower in CY14 (p < 0.001) but not in CY21 and CY28 compared to control (Fig 1F).

### Effect of prepubertal CY exposure on ovulation, fertilization and subsequent embryonic development *in vitro*

Mice exposed to CY showed a reduced ovarian response to superovulation (Fig 1H and S2 Table). No oocytes were recovered in 45% of animals (N = 15 out of 33) in CY14 group. Animals not exposed to CY yielded a total of 50.2 ± 3.2 oocytes per animal, of which 86.3 ± 2.6% oocytes were MII. The total number of oocytes from females responded to superovulation and the maturation rate of retrieved oocytes in CY14 (8.5 ± 1.4 and 73.9 ± 5.7%), CY21 (24.1 ± 2.9 and 79.6 ± 5.9%) and CY28 (26.8 ± 2.1 and 83.4 ± 3.8%) demonstrated a significant decline in the number of oocytes (p < 0.001, Fig 1H) but not in maturation rate compared to control.

**Fig 2:**
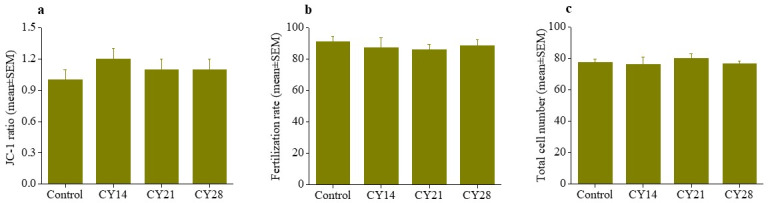
**Effect of prepubertal CY on oocyte competence at 14 weeks of life** (a) mitochondrial activity in MII oocytes, in control (N = 45), CY14 (N = 22), CY21 (N = 36) and CY28 (N = 58) (b) fertilizing ability of the *in vivo* matured MII oocytes in the presence of capacitated caudal spermatozoa from healthy males, in control (N = 270), CY14 (N = 108), CY21 (N = 324) and CY28 (N = 316) oocytes and (c) total cell number (TCN) in expanded and hatched blastocysts at 120 hpi, in control (N = 33), CY14 (16), CY21 (N = 43) and CY28 (N = 33) blastocysts. No significant difference found between the study groups.

Mitochondria in oocytes provide energy for fertilization and subsequent preimplantation development. Here we used JC-1, a mitochondrial specific probe to determine the mitochondrial potential in CY treated animals. JC-1 fluoresces red in the presence of active mitochondria by forming aggregates. In the presence of inactive mitochondria as the probe remains in the monomeric state and fluoresces green (S2 Fig). However, JC-1 ratio (red to green) was comparable across all the groups studied (Fig 2A).

The fertilization ability of MII oocytes derived from CY treated animals was tested by inseminating with caudal spermatozoa collected from healthy males. The fertilization rate did not differ in CY groups compared to control (Fig 2B). However, the ability of the embryos to progress to blastocyst was significantly reduced in all CY groups (Table 2). Of the 246 fertilized eggs in the control group, 87% progressed to blastocyst, and 43.9% embryos hatched at 120 hpi. In CY14, only 55.4% of 92 fertilized eggs formed blastocysts (p < 0.0001) and only 16.3% of embryos demonstrated hatching (p < 0.0001, Table 2). Similarly, lower rates of blastocyst formation and hatching were observed in CY21 (70.7%; p < 0.0001 and 31.5%; p < 0.01, respectively) and CY28 (70.6%: p < 0.0001 and 28.4%: p < 0.001) compared to control. However, the blastocysts were morphologically indistinguishable across the groups, and no difference was found in the total cell number (TCN; Fig 2C).

**Table 2:** Effect of prepubertal CY exposure on preimplantation development of embryos *in vitro*.

Groups	Fertilized oocytes (N)	Preimplantation embryo development rate *in vitro* (%)				
		2 cell at 24 hpi	4 cell at 48 hpi	Morula at 72 hpi	Blastocyst at 120 hpi	Hatched blastocyst at 120 hpi
Control	246	244 (99.2)	228 (92.7)	225 (91.5)	214 (87.0)	108 (43.9)
CY14	92	79 (85.9) ^d^	57 (62.0) ^d^	58 (63.0) ^d^	51 (55.4) ^d^	15 (16.3) ^d^
CY21	270	253 (93.7) ^b^	214 (79.3) ^d^	210 (77.8) ^d^	191 (70.7) ^d^	85 (31.5) ^b^
CY28	275	262 (95.3) ^a^	231 (84.0) ^b^	249 (90.5)	194 (70.6) ^d^	78 (28.4) ^c^

^**a**^p < 0.05,

^**b**^p < 0.01,

^**c**^p < 0.001 and

^**d**^p < 0.0001 vs. control.

### Inner Cell Mass (ICM) proliferation ability but not embryo attachment potential was affected by CY treatment

**Fig 3:**
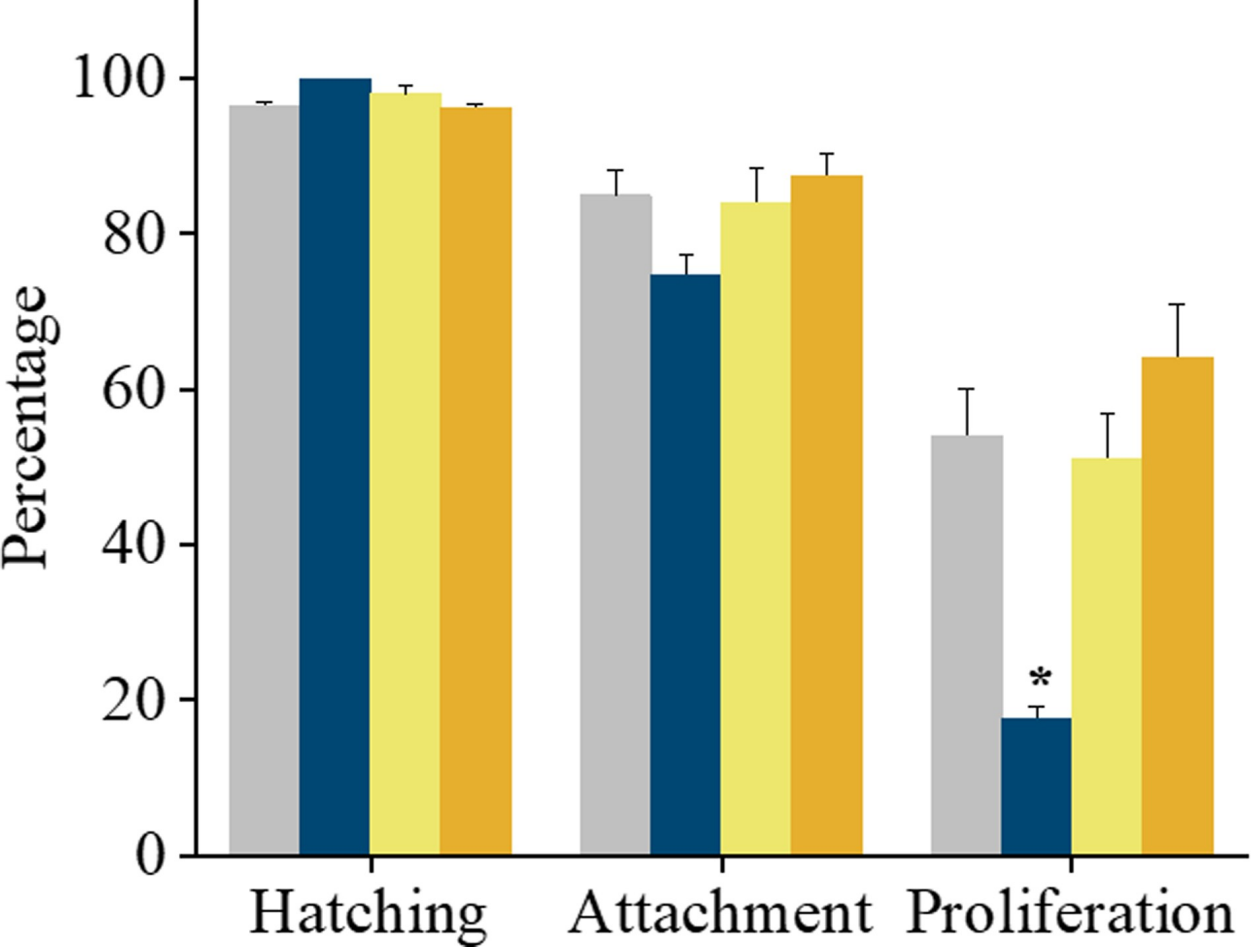
Effect of prepubertal CY exposure on hatching, attachment and proliferation ability of the blastocysts, after ICM culture at 48 h, (i.e. at 168 hpi). The data is presented in mean±SEM, of *in vitro* derived blastocysts hatched, attached or proliferated, in control (N = 112, grey bar), CY14 (N = 23, blue bar), CY21 (N = 87, yellow bar) & CY28 (N = 99, orange bar). **p < 0.05 vs. CY28, N = 3 trial. Bar represents mean and error bar represents standard error of mean.

**Fig 4:**
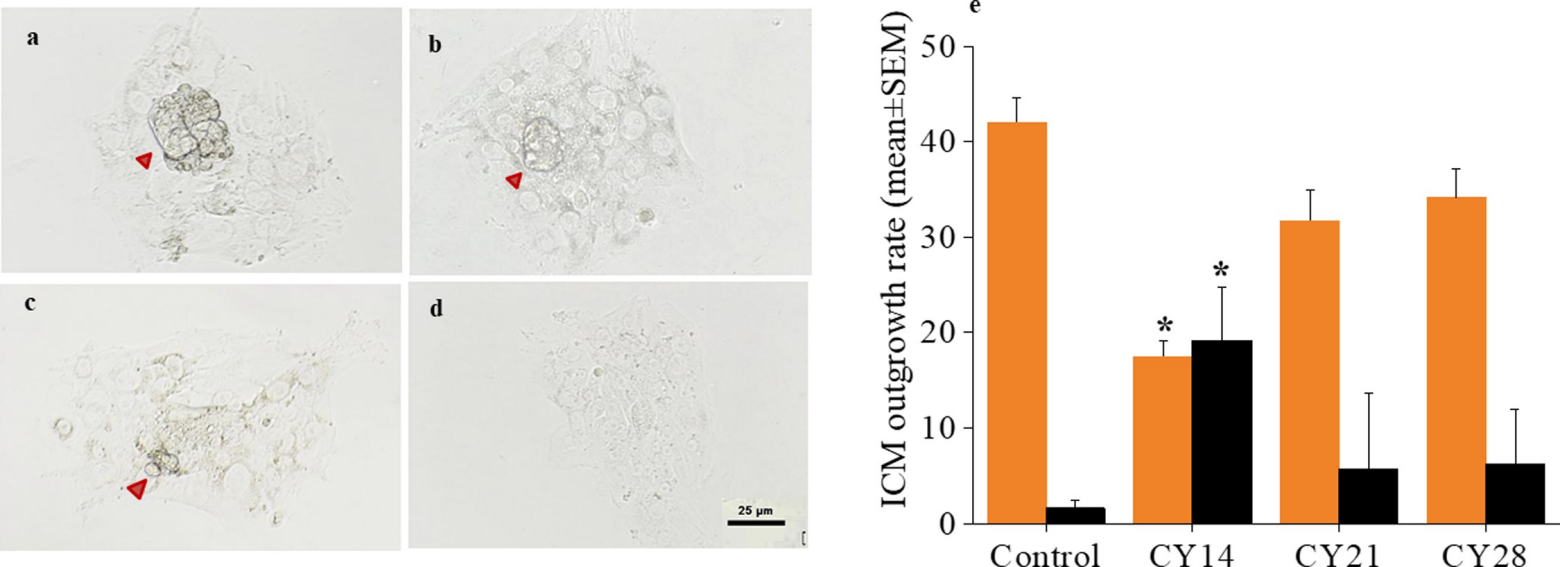
Effect of prepubertal CY exposure on proliferation ability of inner cell mass of blastocysts at 14 weeks of life. (a-d) Representative phase-contrast images of ICM outgrowths at 216 hpi: CIO, LIO, SIO and NIO respectively. Red arrowhead marks the ICM outgrowths (Scale bar 50 μm). (**e**) Proportion of ICM outgrowths of *in vitro* derived blastocysts graded as CIO (orange bar) and NIO (black bar) after culture for 216 hpi *in vitro*, of control (N = 112), CY14 (N = 23), CY21 (N = 87) & CY28 (N = 99) transferred blastocysts. *p < 0.05, vs. control, N = 3 trial. Bar represents mean and SEM is represented by error bar.

As blastocysts derived from CY treated animals were morphologically similar, their ability to attach to gelatin coated plates and to proliferate *in vitro* was tested at 120 hpi. Hatched blastocysts attain adhesive capacity by 24h of *in vitro* culture. The hatching potential and attachment ability of blastocysts transferred to gelatin coated plates did not differ significantly between the control and CY groups. However, after culture for 48 h i.e. at 168 hpi, the attached blastocysts from CY14 showed significant delay in ICM cell proliferation *in vitro* (17.5±1.5 vs. 64.0±6.9 in CY28: p < 0.05, Fig 3), whereas proliferation was unaffected in CY21 (51.1±5.9) and CY28 compared to control (53.9±6.1) group. ICM outgrowths were graded morphologically at 96 h, i.e. 216 hpi (Fig 4A–4D). The number of embryos with completely developed outgrowths (CIO) was significantly lower in CY14 (17.5±1.7; p < 0.05) and the proportion with no outgrowth was higher in CY14 (19.2±5.6, p < 0.05) compared to control (Fig 4E). However, the ICM and TE area and the ICM to TE ratio did not significantly differ across the study groups (S3 Table).

## Discussion

The current study indicates that the early prepubertal rodent ovary is more vulnerable to CY induced reproductive toxicity than the other age groups tested. The extent of the impairments observed in this study include an expected reduction in the ovarian reserve, but also clearly show compromised functional ability of the surviving female germ cells when animals attained adulthood, with greater impairment following CY exposure at younger ages, thus despite a longer period for potential recovery.

Cytotoxic effects of CY on normal proliferative cells in mice [23], including human ovarian cells [24] are well-known. It has been shown in the murine model that primordial follicles are also sensitive to CY toxicity, as are antral and growing follicles [5–7,9,25]. The diminished primordial follicle numbers in the present study show that prepubertal ovaries are also sensitive to CY induced follicular depletion. Consistent with many studies showing reduced AMH levels after childhood cancer therapy [26–28], in the present study AMH was reduced in CY-treated animals in adulthood, most markedly in CY14. In keeping with these findings, fewer GV oocytes were obtained after superovulation from CY-exposed animals, again with the most marked reduction being in CY14, treated at the youngest age.

Preimplantation mouse embryos show unique stage dependent checkpoint responses to genetic insults whereby p53 mediated checkpoint is active soon after fertilization followed by several other checkpoint mechanisms to protect embryos [29–31]. The integrity of the genome is constantly at risk due to the errors intrinsic to DNA replication [29]. It is important to note that embryos have an efficient DNA damage response pathway after implantation, and this is crucial for the proper development of the organism during this period [32]. The ability of oocytes to undergo *in vitro* fertilization was not significantly altered in CY exposed animals in comparison to control. However, preimplantation developmental competence was significantly reduced in embryos derived from CY14. Importantly, as also shown by the ovarian and oocyte data, all these effects were more marked in the animals treated at the youngest age, with reduced impact in more mature animals. In rodents, *in vitro* embryonic block at the 2-cell stage is a common phenomenon and causes are multifactorial. Since the embryo progression from 2-cell to blastocyst stage did not vary significantly across CY exposed groups (Table 2), it is possible that CY embryos had higher tendency to experience 2-cell block.

Mouse preimplantation embryos carrying induced DNA lesions are morphologically indistinguishable from control embryos at the blastocyst stage [33], and consistent with this, embryos across the groups were morphologically normal in the present study. Due to limited damage sensing and repair ability, preimplantation embryos have the tendency to progress beyond the blastocyst stage despite carrying genetic damage. Hence, we considered assessing their functional ability beyond the blastocyst stage using the *in vitro* ICM proliferation ability assay. Hatched mouse blastocysts attain adhesive capacity by 24 h of *in vitro* culture [34]. In our study, the potential of hatched blastocysts to attach was similar in control and CY groups. However a significant reduction in blastocyst outgrowths was observed in CY14 embryos. *In vivo* studies have shown that excessive apoptosis in the ICM compartment of blastocysts may lead to post implantation demise, possibly due to inadequate number of cells available to form cell lineages [35]. While the present data cannot mimic the *in vivo* developmental competence of the embryo, investigation of the reproductive outcome and transgenerational risk associated with CY exposure will be important in future studies to address the risk associated with CY treatment in oncological patients.

Results from the present study clearly show that in addition to effects on loss of ovarian follicles, the administration of CY at 14 day of life has marked effects on the subsequent ability of oocytes to mature and fertilize, on normal early embryonic development, and on the formation of ICM outgrowths. All these adverse effects were more marked in animals exposed at the youngest age. These findings may at least in part underlie the reduced fertility of women despite retained ovarian function after exposure to chemotherapy in childhood. Overall, the study provides insight into the need for fertility preservation in young prepubertal age patients before undergoing cytotoxic chemotherapy, and the need for ongoing surveillance of their children [1].

## Supporting information

S1 Table Effect of prepubertal CY exposure on ovary, spleen, kidney and liver (relative) weight at 14 weeks of life. ^**a**^p < 0.05, ^**b**^p < 0.01 and ^**c**^p < 0.001 vs. control.
(DOCX)

S2 Table Effect of prepubertal CY exposure on oocyte number and quality, post-superovulation at 14 weeks of life. ^**a**^p < 0.05, ^**b**^p < 0.001 vs. control.
(DOCX)

S3 Table Effect of prepubertal CY exposure on the ability of inner cell mass to proliferate *in vitro* at 216 hpi. No statistical significance difference found between the groups.
(DOCX)

S1 Fig Effect of prepubertal CY exposure on ovarian follicle reserve at 14 weeks of life. Mean (±SE) number of primordial (grey bar), primary (purple bar), secondary (yellow bar) and antral follicles (orange bar) examined in Control (N = 9), CY14 (N = 7), CY21 (N = 4) & CY28 (N = 6). **p < 0.01 vs. control.
(TIF)

S2 Fig Representative images of JC-1 monomers and JC-1 aggregates in denuded MII oocytes captured under a fluorescence microscope, (a) Uniform distribution of active mitochondria, (b) peripheral distribution of active mitochondria, and (c) inactive mitochondria, at 40X magnification.
(TIF)
