# Supplementary material for: Early prepubertal cyclophosphamide exposure in mice results in long-term loss of ovarian reserve, and impaired embryonic development and blastocyst quality
Source: PLoS One. 2020 Jun 23;15(6):e0235140. doi: 10.1371/journal.pone.0235140 (PMC7310698; doi:10.1371/journal.pone.0235140)
Supplement: S1 Table — ap < 0.05, bp < 0.01 and cp < 0.001 vs. control. (DOCX) [file pone.0235140.s001.docx]

Supplementary data Table S1: Effect of prepubertal CY exposure on ovary, spleen, kidney and liver (relative) weight at 14 weeks of life.

| Groups | Number of females | Body weight  (g) | Ovary  (mg) | Uterine horn  (mg) | Spleen  (mg) | Kidney  (mg) | Liver  (mg) |
| --- | --- | --- | --- | --- | --- | --- | --- |
| Control | 12 | 27.9 ± 0.4 | 13.8 ± 0.5 | 117.3 ± 9.2 | 151.3 ± 8.8 | 171.4 ± 4.8 | 1452.3 ± 36.0 |
| CY14 | 33 | 24.6 ± 0.6 **^b^** | 5.0 ± 0.5 **^c^** | 78.6 ± 4.0 **^c^** | 141.4 ± 5.4 | 164.2 ± 3.6 | 1629.2 ± 24.4 **^b^** |
| CY21 | 16 | 24.7 ± 0.6 **^b^** | 9.3 ± 0.8 **^c^** | 96.4 ± 6.0 | 158.8 ± 7.4 | 163.8 ± 4.7 | 1607.5 ± 22.5 **^a^** |
| CY28 | 14 | 25.2 ± 0.6 **^a^** | 10.1 ± 0.6 **^b^** | 76.2 ± 5.2 **^c^** | 182.9 ± 13.4 | 170.0 ± 6.7 | 1589.0 ± 40.5 |

**^a^**p < 0.05, **^b^**p < 0.01 and **^c^**p < 0.001 vs. control
