# Supplementary material for: Early prepubertal cyclophosphamide exposure in mice results in long-term loss of ovarian reserve, and impaired embryonic development and blastocyst quality
Source: PLoS One. 2020 Jun 23;15(6):e0235140. doi: 10.1371/journal.pone.0235140 (PMC7310698; doi:10.1371/journal.pone.0235140)
Supplement: S2 Table — ap < 0.05, bp < 0.001 vs. control. (DOCX) [file pone.0235140.s002.docx]

Supplementary data Table S2: Effect of prepubertal CY exposure on oocyte number and quality, post-superovulation at 14 weeks of life.

| Group | Number of females | Oocytes per female | Total  oocytes | MII  oocytes | MII oocytes per female | Fragmented oocytes | Maturation rate |
| --- | --- | --- | --- | --- | --- | --- | --- |
| Control | 6 | 50.2 ± 3.2 | 301 | 270 | 45.0 ± 3.0 | 31 | 86.3 ± 2.6 |
| CY14 | 18 | 8.5 ± 1.4 **^b^** | 153 | 108 | 6.0 ± 1.1 **^b^** | 45 | 73.9 ± 5.7 |
| CY21 | 16 | 24.1 ± 2.9 **^b^** | 385 | 324 | 20.3 ± 3.0 **^a^** | 61 | 79.6 ± 5.9 |
| CY28 | 14 | 26.8 ± 2.1 **^b^** | 375 | 316 | 22.6 ± 2.1 | 59 | 83.4 ± 3.8 |

**^a^**p < 0.05, **^b^**p < 0.001 vs. control
