# Supplementary material for: Early prepubertal cyclophosphamide exposure in mice results in long-term loss of ovarian reserve, and impaired embryonic development and blastocyst quality
Source: PLoS One. 2020 Jun 23;15(6):e0235140. doi: 10.1371/journal.pone.0235140 (PMC7310698; doi:10.1371/journal.pone.0235140)
Supplement: S3 Table — No statistical significance difference found between the groups. (DOCX) [file pone.0235140.s003.docx]

Supplementary data Table S3: Effect of prepubertal CY exposure on the ability of inner cell mass to proliferate *in vitro,* at 216 hpi.

| Groups | Total transfer | Grade 3 | ICM area of CIO (µm^2^) | ICM/TE ratio  (grade 3) | Grade 2 | ICM area of  LIO (µm^2^) | ICM/TE ratio  (grade 2) | Grade 1 | ICM area of  SIO (µm^2^) | ICM/TE ratio  (grade 1) |
| --- | --- | --- | --- | --- | --- | --- | --- | --- | --- | --- |
| Control | 122 | 47 | 21715 ± 1064 | 0.109 ± 0.004 | 39 | 10248 ± 458 | 0.072 ± 0.007 | 8 | 3221 ± 482 | 0.019 ± 0.003 |
| CY14 | 23 | 4 | 27988 ± 4844 | 0.118 ± 0.006 | 7 | 11278 ± 912 | 0.075 ± 0.01 | 2 | 3618 ± 150 | 0.023 ± 0.002 |
| CY21 | 87 | 28 | 22821 ± 1333 | 0.114 ± 0.008 | 34 | 9798 ± 503 | 0.066 ± 0.007 | 5 | 3911 ± 670 | 0.026 ± 0.007 |
| CY28 | 99 | 34 | 22324 ± 1122 | 0.108 ± 0.006 | 32 | 10052 ± 531 | 0.067 ± 0.006 | 15 | 2609 ± 321 | 0.017 ± 0.002 |

No statistical significance difference found between the groups.
