# Supplementary figures and images for: Early prepubertal cyclophosphamide exposure in mice results in long-term loss of ovarian reserve, and impaired embryonic development and blastocyst quality
Source: PLoS One. 2020 Jun 23;15(6):e0235140. doi: 10.1371/journal.pone.0235140 (PMC7310698; doi:10.1371/journal.pone.0235140)

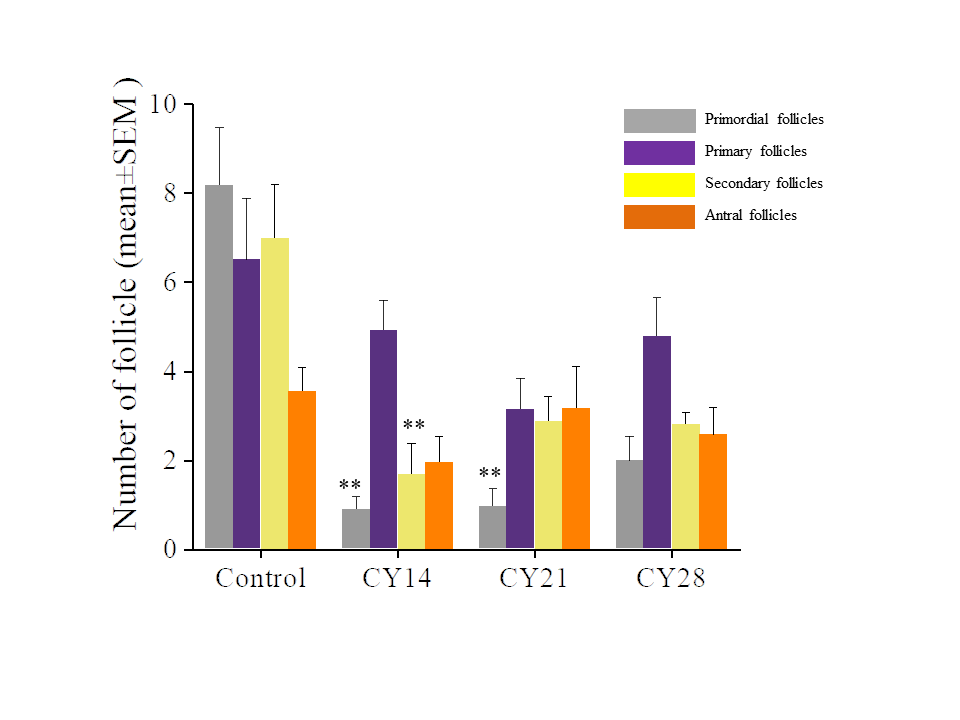

Supplement: S1 Fig — Mean (±SE) number of primordial (grey bar), primary (purple bar), secondary (yellow bar) and antral follicles (orange bar) examined in Control (N = 9), CY14 (N = 7), CY21 (N = 4) & CY28 (N = 6). **p < 0.01 vs. control. (TIF) [file pone.0235140.s004.tif]

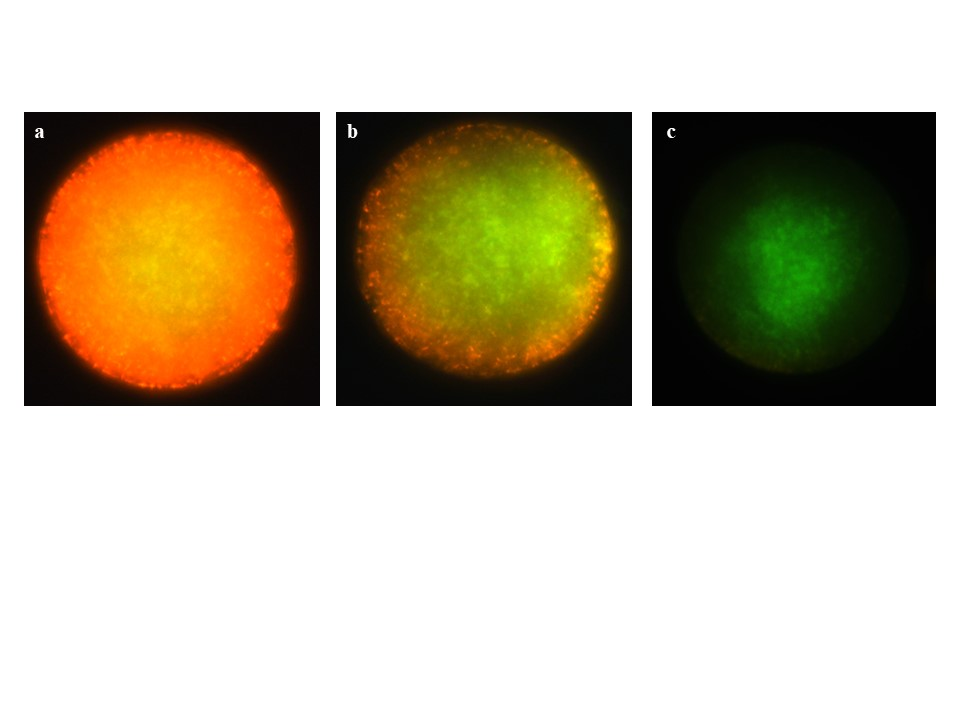

Supplement: S2 Fig — Representative images of JC-1 monomers and JC-1 aggregates in denuded MII oocytes captured under a fluorescence microscope, (a) Uniform distribution of active mitochondria, (b) peripheral distribution of active mitochondria, and (c) inactive mitochondria, at 40X magnification. (TIF) [file pone.0235140.s005.tif]
